# Activation of circulating TFH17 cells associated with activated naive and double negative 2 B cell expansion, and disease activity in systemic lupus erythematosus patients

**DOI:** 10.1186/s13075-024-03394-7

**Published:** 2024-09-11

**Authors:** Tipanan Khunsri, Pongsakorn Thawornpan, Pachara Tianpothong, Thanitta Suangtamai, Pintip Ngamjanyaporn, Chaniya Leepiyasakulchai, Kittikorn Wangriatisak, Prapaporn Pisitkun, Patchanee Chootong

**Affiliations:** 1 Department of Clinical Microbiology and Applied Technology, Faculty of Medical Technology, Mahidol University, Bangkok, Thailand; 2 Division of Allergy, Immunology and Rheumatology, Department of Medicine, Faculty of Medicine, Ramathibodi Hospital, Mahidol University, 270 Rama 6 Road, Ratchathewi, Bangkok, Thailand

**Keywords:** Systemic lupus erythematosus, Activated naïve B cells, Double negative 2 B cells, Follicular helper T cells

## Abstract

**Background:**

Systemic lupus erythematosus (SLE) is the quintessential autoimmune disease, as it is characterized by hyperactivity of CD4^+^ T cells and subsequently drives lupus pathology. Follicular helper T (TFH) cells play an important role in B cell maturation and antibody production. However, which specific subset of cTFH cells drives B cell function and contributes to the development of anti-dsDNA antibodies and SLE pathogenesis remains unclear.

**Methods:**

Peripheral blood mononuclear cells from SLE patients with inactive (*n* = 11) and active (*n* = 21) were used to determine and detect frequencies and phenotypes of circulating TFH cells (cTFH), memory cTFH, and B cell subsets. The correlations among cTFH cell subsets and phenotypes, B cell subsets, anti-dsDNA autoantibodies, and clinical parameters were analyzed.

**Results:**

In subjects with active SLE, cTFH1 and cTFH17 cells were significantly expanded and activated. These expanded cTFH cells expressed memory phenotypes; cTFH1 cells were predominantly central memory (CM) type, while cTFH17 cells were largely effector memory (EM) type. Phenotyping B cell subsets in these patients showed increased frequencies of aNAV and DN2 B cells. Clinically, ICOS^+^ cTFH1, ICOS^+^ cTFH17 cells, and SLEDAI-2k scores were found to be correlated. Analysis of cTFH-B cell relationship revealed positive correlations among ICOS^+^ cTFH1 cells, aNAV B cells, and anti-dsDNA antibodies. Activation of ICOS^+^ cTFH17 cells was significantly related to the expansion of aNAV and DN2 B cells. The presence of CM cells in cTFH1 and cTFH17 subsets was correlated with aNAV and DN2 B cell frequencies.

**Conclusion:**

SLE cTFH cells were found to be polarized toward cTFH1 and cTFH17 cells; activation of these cTFH subsets was significantly associated with disease activity score, aNAV, DN2 B cell expansion, and anti-dsDNA antibody level. Thus, the interactions among cTFH1, cTFH17, and B cells likely contribute to the development of autoantibodies and the pathogenesis in SLE.

**Supplementary Information:**

The online version contains supplementary material available at 10.1186/s13075-024-03394-7.

## Background

Systemic lupus erythematosus (SLE) is a systemic autoimmune disease characterized by a loss of immunologic tolerance. Recognition of self-antigens causes abnormal immune responses that result in the generation of autoantibodies by B cells [1]. Among antibodies that react with self-antigens, anti-dsDNA antibodies are unique in their association with a pathological state [2, 3]. These antibodies bind to self-antigens and form immune complexes which accumulate, migrate through the bloodstream, and activate complement and cytokines, leading to multiorgan inflammation and tissue damage [3]. Of note, activated naive (aNAV) B cells were implicated in the secretion of anti-dsDNA antibodies. An expansion of DNA autoreactive aNAV B cells was positively related to disease activity suggesting a pathogenic role of aNAV B cell in SLE by the active autoantibody production [4]. However, the mechanisms which drive these autoreactive B cells to generate anti-dsDNA antibodies are still unclear. A greater understanding of the mechanism of induction of dsDNA-specific B cells may allow the use of these cells as biomarkers of disease activity in SLE patients.

It has been reported that T cell-dependent antibody responses are the main drivers of SLE pathogenesis, both in humans [5, 6] and lupus-prone mice [7]. The activation of B cells in a T-dependent manner produces self-antigen-specific IgG that plays a role in tissue inflammation and pathology [8–10]. Follicular helper T (TFH) cells, displaying the CXCR5^+^PD1^+^ phenotype support germinal center (GC) formation and maintenance of humoral immunity [11]. These cells express co-stimulatory molecules [CD40 ligand, inducible co-stimulator (ICOS), PD-1, and CD28] for B cells, and secrete IL-4, IL-10, and IL-21 cytokines that promote the differentiation and class-switching of these cells [11, 12]. To assess TFH responses in human, analysis of circulating CD4^+^ T cells expressing CXCR5 [circulating TFH (cTFH) cells] has been performed. These cTFH cells are heterogeneous and generate cytokine-skewed immune responses. Three district subsets have been identified and are classified based on differences in the level of expression of the chemokine receptors: CXCR3 and CCR6, cTFH1 (CXCR3^+^CCR6^−^), cTFH2 (CXCR3^−^CCR6^−^) and cTFH17 (CXCR3^−^CCR6^+^) [13].

In SLE patients, it has been reported that circulating cTFH cells (CXCR5^+^ICOS^+^PD1^+^) are expanded [14–16], and positively correlated with disease activity [15, 17]. Among cTFH subsets, an expansion of TFH-like Th1 cells (CXCR5^+^CXCR3^+^) is found in Asian patients [18]. An analysis of the cTFH and B cell relationship demonstrated that the frequency of cTFH2 is statistically correlated with anti-dsDNA autoantibodies and plasmablasts [19], although the cTFH2 frequency did not statistically differ from that in healthy subjects [15]. This indicates the role of cTFH2 cells in supporting humoral immunity. As for cTFH17 cells, an increased frequency was shown in active SLE patients [20], while the percentage of these cells was reduced in patients with lupus nephritis [21]. A few reports indicate that the frequency of cTFH17 is not positively related to disease activity score and plasmablast frequency [19]. Together, previous studies provide evidence that SLE induces cTFH cell responses by changing the proportions of the subsets and phenotypes. However, the mechanisms behind these associations of cTFH cell subsets with disease activity and aberrant B cell responses remain unclear. Elucidating which specific subset or phenotype of cTFH cells plays the major role in driving B cells to produce the autoantibodies leading to SLE development could be useful in identifying targets for therapeutic intervention.

In this study, we aimed to identify a subset or phenotype of cTFH cells that are significantly correlated with the B cell responses producing anti-dsDNA autoantibodies and, thus, disease activity in SLE patients. We comprehensively analyzed the phenotypes of cTFH cell subsets and memory cTFH cells and evaluated the associations of cTFH subsets, memory cTFH cells, B cell subsets, and anti-DNA autoantibodies with clinical parameters.

## Methods

### Subjects and study design

A total 32 SLE patients with ages above 18 years were enrolled from the Department of Medicine, Ramathibodi Hospital, Bangkok, Thailand. All patients met the Systemic Lupus International Collaborating Clinics (SLICC) 2012 criteria or the American College of Rheumatology (ACR) criteria for SLE diagnosis and classification. Disease activity is evaluated with SLE disease activity index (SLEDAI-2k) with a SLEDAI score of 0 indicating inactive disease (*n* = 11) and a SLEDAI score ≥1 indicating active disease (*n* = 21) [22]. Patients received treatment according to disease activity and degree of organ damage. All patients were without infection, tumors, and other autoimmune diseases. Sixteen adults who lived in Bangkok, Thailand, and were without SLE were recruited as healthy controls (HCs). Written informed consent was obtained from all subjects. The study was approved by the Ethical Committee of Mahidol University (approval number, MURA 2015/731). The clinical parameters and medications of subjects are summarized in Supplementary Table 1.

### Antibodies and reagents

The following antibodies (all from Biolegend, San Diego, CA, USA) were used for phenotyping cTFH cell subsets: AF700-CD3 clone OKT3, PerCP/Cy5.5-CD4 clone RPA-T4, PE-CXCR5 clone J252D4, APC-PD-1 clone EH12.2H7, APC/Fire750-CXCR3 clone G025H7 and PE/Cy7-CCR6 clone G034E3; and for memory phenotypes: AF700-CD3 clone OKT3, PerCP/Cy5.5-CD4 clone RPA-T4, PE-CXCR5 clone J252D4, APC-PD-1 clone EH12.2H7, APC/Fire750-CXCR3 clone G025H7, PE/Cy7-CCR6 clone G034E3, FITC-CCR7 clone G043H7, PE/Dazzle-CD45RA clone HI100 and FITC-ICOS clone C398.4 A. For B cell staining, the following antibodies (all from Biolegend) were used: FITC-CD19 clone HIB19, APC/Fire750-CD27 clone M-T271, PerCP/Cy5.5-CD11c clone Bu15, AF700-CXCR5 clone J252D4, PE/Dazzle-IgD clone IA6-2, and PE/Cy7-IL-21 receptor (IL-21R) clone 17A12.

### Cell staining and flow cytometry

Fresh human heparinized blood from SLE patients (*n* = 32) was used to isolate peripheral blood mononuclear cells (PBMCs) by density-gradient centrifugation with Ficoll-Hypaque solution (Lymphoprep™ STEMCELL Technologies, Vancouver, BC, Canada). The PBMCs were washed three times with phosphate-buffered saline (PBS), stained with fluorochrome antibodies, and analyzed by flow cytometry. The cTFH cells were identified as CXCR5^+^PD1^+^ cells gated on CD4^+^ T cells, then were classified into 4 distinct subsets using CXCR3 and CCR6 markers: cTFH1 (CXCR3^+^CCR6^−^), cTFH2 (CXCR3^−^CCR6^−^), cTFH17 (CXCR3^−^CCR6^+^) and cTFH17.1 (CXCR3^+^CCR6^+^) [12, 23]. In addition, the phenotypes of memory cTFH cells were defined based on CCR7 and CD45RA markers [24, 25]. Four subsets were classified; (1) naive cells; CCR7^+^CD45RA^+^, (2) central memory (CM) T cells; CCR7^+^CD45RA^−^ cells, (3) effector memory (EM) T cells; CCR7^−^CD45RA^−^ cells, and (4) terminally differentiated effector memory (TEMRA) T cells; CCR7^−^CD45RA^+^ cells were classified.

To phenotype B cell subpopulations, total B cells were gated from CD19^+^ and classified into CD27^+^IgD^+^ unswitched memory B cells (USW), CD27^+^IgD^+^ switched memory B cells (SWM), CD27^−^IgD^+^ naïve B cells (NAV), and CD27^−^IgD^−^ double negative B cells (DN). Based on CD11c and CXCR5 expression levels, NAV cells were classified into CD11c^+^CXCR5^−^ resting naïve B cells (rNAV) and CD11c^−^CXCR5^+^ activated naïve B cells (aNAV). The DN cells were distinguished as CD11c^+^CXCR5^−^ double negative 1 (DN1) or CD11c^−^CXCR5^+^ double negative 2 (DN2). Data acquisition was performed with the FACSCanto cytometer (BD FACSCanto II, Becton-Dickinson Immunocytometry Systems, San Jose, CA, USA). The data were analyzed with FlowJo (Tree Star) software.

### Statistical analysis

Data were analyzed using GraphPad Prism software version 9 (GraphPad Software, San Jose, CA, USA). Statistical testing was performed using one-way ANOVA and Dunn’s multiple comparison tests. Group differences with *p*-values less than 0.05 were considered statistically significant. For correlation analyses, the corrplot package in R version 0.90 was used to plot the graph, and data was clustered by ward.D2 hierarchical agglomerative clustering method and significance of correlations was calculated using Spearman’s correlation coefficient (r).

## Results

### Expansion and activation of cTFH1 and cTFH17 cells

**Fig. 1:**
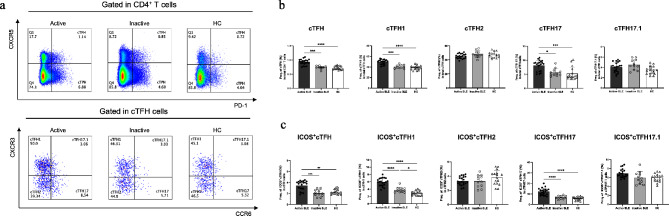
The distribution of cTFH cell subsets in SLE patients. (**a**) Representative flow cytometry gating strategy of cTFH cells from one SLE patient and one HC. Four subsets were gated: cTFH1 (CXCR3^+^CCR6^−^), cTFH2 (CXCR3^−^CCR6^−^), cTFH17 (CXCR3^−^CCR6^+^), and cTFH17.1 (CXCR3^+^CCR6^+^) (**b**) The frequency of total cTFH cells and their subsets in active (*n* = 21) and inactive SLE patients (*n* = 11) compared to HCs (*n* = 16). (**c**) Frequency of ICOS-expressing cTFH cell subsets in the SLE patients and HCs. Bars represent the median with interquartile range; *p* values were determined by the one-way ANOVA: * *p* < 0.05, ** *p* < 0.01, *** *p* < 0.001, **** *p* < 0.0001, and ns: not significant

To identify specific cTFH cell subsets that are involved in SLE pathogenesis, we assessed the total number of cTFH cells and their subsets. Based upon the differential expression of CXCR3 and CCR6 on CD4^+^CXCR5^+^PD-1^+^ T cells, four subsets were identified: CXCR3^+^CCR6^−^(cTFH1) cells, CXCR3^−^CCR6^−^ (cTFH2) cells, CXCR3^−^CCR6^+^ (cTFH17) cells and CXCR3^+^CCR6^+^ (cTFH17.1) (Fig. 1a). The frequency of each cTFH subset and ICOS^+^ cTFH cells in SLE patients and HCs was summarized in Table 1. The frequency of total cTFH cells was significantly (*p* < 0.0001) elevated in SLE patients compared to HCs. Subjects with active disease had a higher frequency of total cTFH than both those with inactive disease and HCs (*p* < 0.0001) (Fig. 1b). A further analysis of cTFH subsets demonstrated a significantly increased frequency of cTFH1 (*p* < 0.001) and cTFH17 cells (*p* < 0.001) in subjects with active disease, compared to those inactive. However, the frequency of cTFH2 and cTFH17.1 cells in SLE patients was not statistically different from that of HCs (Fig. 1b). The frequency of cTPH cells (CD4^+^CXCR5^−^PD-1^+^) was also detected in our SLE subjects. A significant increase of cTPH1 and cTPH17 was found in active SLE, compared to HCs (Supplementary Fig. 1).

**Table 1:** The frequency of cTFH cell subsets in active (*n* = 21), inactive SLE (*n* = 11), and HCs (*n* = 16)

Parameters	Active SLE Median [IQR]	Inactive SLE Median [IQR]	Healthy controls Median [IQR]	*p*-value
Total cTFH	0.94 [0.89–1.01]	0.77 [0.70–0.80]	0.74 [0.71–0.76]	< 0.0001
cTFH1	50.30 [45.70-52.95]	40.00 [37.80–43.10]	38.98 [36.28–41.71]	< 0.001
cTFH17	8.71 [ 6.07–9.33]	5.12 [4.75–7.97]	4.67 [3.78–6.39]	< 0.001
ICOS^+^ cTFH1	5.93 [5.01–6.95]	3.60 [3.18–4.13]	2.75 [2.25–5.36]	< 0.0001
ICOS^+^cTFH17	12.20 [9.26–14.18]	6.90 [5.67-8.00]	6.67 [5.60–7.30]	< 0.0001
cTFH1-CM	49.80 [45.8-52.15]	37.00 [34.5-41.04]	24.25 [20.0-26.4]	< 0.001
cTFH2-CM	38.73 [31.57–48.47]	35.45 [32.18–39.70]	32.70 [23.93–38.23]	ns
cTFH17-CM	48.70 [44.74–52.43]	47.00 [32.35–40.38]	37.85 [30.60–49.50]	< 0.01
cTFH1-EM	31.60 [30.31–34.50]	30.71 [29.06–34.11]	30.35 [27.77–33.10]	ns
cTFH2-EM	33.00 [29.03–35.32]	32.71 [32.00-35.30]	32.35 [31.32-36.00]	ns
cTFH17-EM	41.60 [38.32–45.55]	39.00 [31.72–43.41]	36.50 [32.47–38.63]	< 0.01

To assess the activation status of the expanded cTFH1 and cTFH17 cells, we determined the expression of ICOS molecules on cell surfaces (Supplementary Fig. 2). The ICOS-expressing cells, in both cTFH1 and cTFH17 cells of SLE patients were significantly more frequent than in HCs (Fig. 1c). In active patients, ICOS expression was significantly increased in cTFH1 (*p* < 0.0001) and cTFH17 (*p* < 0.0001) cells compared to inactive subjects and HCs (Fig. 1c).

### The cTFH1 cells expressed CM phenotypes, while memory cTFH17 cells were altered into effector phase in active SLE patients

**Fig. 2:**
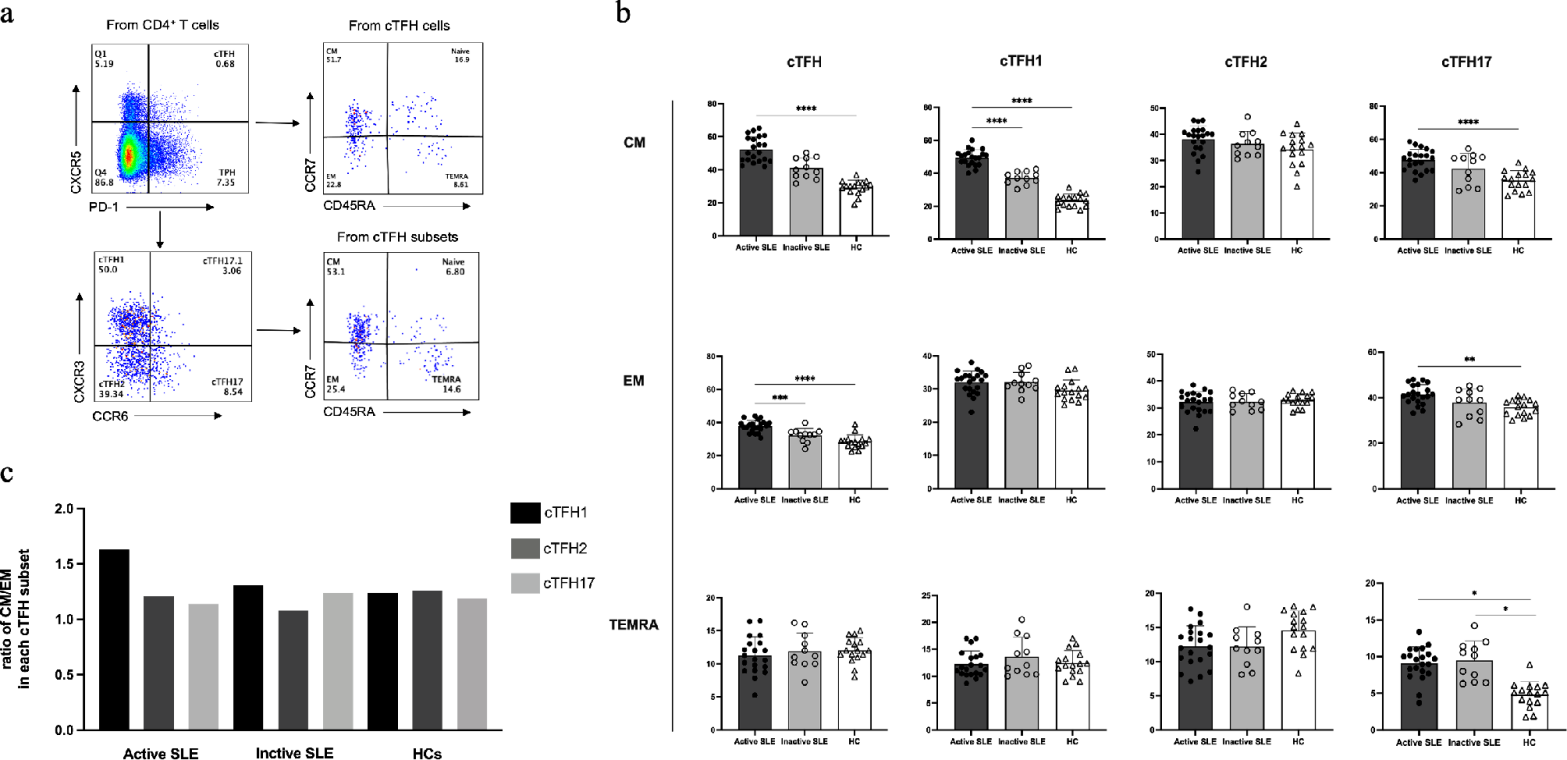
The distribution of CM and EM cells in each subset of memory cTFH cells. (**a**) Representative flow cytometry gating strategy of an SLE subject. Memory cTFH cells were classified and grouped as central memory (CM; CCR7^+^CD45RA^−^), effector memory (EM; CCR7^−^CD45RA^−^), TEMRA (CCR7^−^CD45RA^+^), or naive (CCR7^+^CD45RA^+^). (**b**) The frequency of total cTFH cells and their subsets (cTFH1, cTFH2, and cTFH17) in active (*n* = 21) and inactive SLE patients (*n* = 11) compared to HCs (*n* = 16). (**c)** The ratio of CM and EM **(**CM**/**EM**)** in each cTFH subset in SLE patients and HCs. *p* values were determined by the one-way ANOVA: * *p* < 0.05, ** *p* < 0.01, *** *p* < 0.001, **** *p* < 0.0001, and ns: not significant

To assess the development of memory TFH cells and its subsets in SLE patients, the phenotypes of CM, EM, TEMRA, and naive cTFH cells were analyzed (Fig. 2a). The frequency of these memory cTFH cell subsets in SLE patients and HCs were summarized in Table 1. For CM cells, cTFH1-CM in actinve patients were significantly higher than subjects and HCs (*p* < 0001) (Fig. 2b). Also, there was an increased frequency of cTFH17-CM cells in active, compared to both inactive and HCs (Fig. 2b). In contrast, there were no statistical differences in cTFH2-CM cells among active, inactive disease subjects and HCs (Fig. 2b). The phenotyping of EM cells showed that cTFH17-EM had a higher frequency in active patients, compared to HCs (*p* < 0.01) (Fig. 2b). However, frequency of cTFH1-EM and cTFH2-EM in patients did not greatly differ from HCs (Fig. 2b). In addition, TEMRA cells showed an elevation of cTFH17-TEMRA in both active (*p* < 0.05) and inactive patients (*p* < 0.05) compared to baseline in HCs (Fig. 2b).

To observe the changing distribution of memory cTFH phenotype, we analyzed the ratio of CM and EM (CM/EM) in each cTFH subset. In active SLE subjects, the highest ratios of CM/ EM were in cTFH1 (CM/EM = 1.63), while the CM/EM ratio in cTFH17 was the lowest (CM/EM = 1.14). The CM/EM ratio in cTFH2 cells was 1.21. The higher CM/EM ratio of cTFH1 and cTFH17 cells was detected in inactive SLE, compared to CM/EM ratio of cTFH2. (Fig. 2c).

### The increased cTFH1 and cTFH17 cells and their memory phenotype were positively related to disease activity scores

**Fig. 3:**
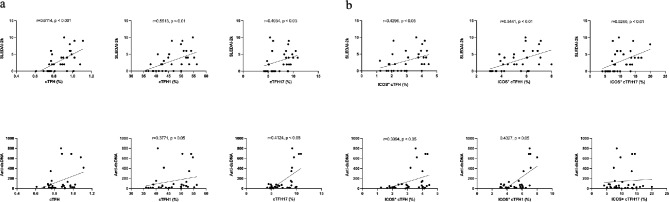
The correlation of cTFH subsets, anti-dsDNA antibodies, and disease activities. The associations of different cTFH subsets, disease activity, and autoantibodies were assessed in SLE patients (*n* = 32). (**a**) The percentage of cTFH subsets, SLEDAI-2k scores, and anti-dsDNA antibody levels. (**b**) The percentage of ICOS^+^ cTFH cells, SLEDAI-2k scores, and anti-dsDNA antibody levels. Spearman’s correlation coefficient (r) and p-value (p): * *p* < 0.05, ** *p* < 0.01, *** *p* < 0.001, **** *p* < 0.0001, and ns: not significant

To further address the contribution of cTFH1 and cTFH17 cells in SLE pathogenesis, we explored the relationship between activation of cTFH cells and disease activity (SLEDAI-2k score), and anti**-**dsDNA autoantibody. cTFH1 and cTFH17 cells were expanded, and positively related to both disease activity scores (cTFH1 *r* = 0.55, *p* < 0.01; cTFH17 *r* = 0.41, *p* < 0.05), and anti-dsDNA antibody levels (cTFH1 *r* = 0.38 *p* < 0.05; cTFH17 *r* = 0.41, *p* < 0.05) (Fig. 3a). An upregulation of ICOS in cTFH1 (*r* = 0.54, *p* < 0.01) and cTFH17 (*r* = 0.53, *p* < 0.01) showed significant correlations with disease activity scores (Fig. 3b). However, only ICOS^+^ cTFH1 cells were positively correlated with anti-dsDNA antibodies (*r* = 0.44, *p* < 0.05) (Fig. 3b). Additionally, there were no positive correlations of cTFH1, cTFH17, ICOS^+^ cTFH1, ICOS^+^ cTFH17 cells with other clinical parameters (complement level C3, C4, ESR, and serum albumin) (Supplement Figs. 3 and 4).

**Fig. 4:**
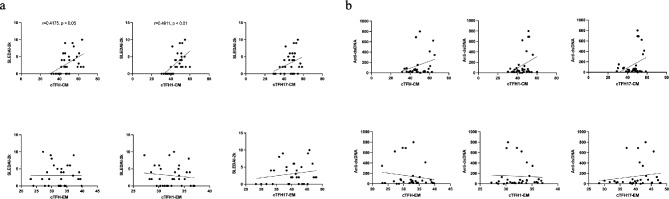
The correlation of memory cTFH-CM, cTFH-EM cells, and disease activities. The Spearman approach was used to estimate the correlation of memory cTFH subsets, disease activity, and autoantibodies in SLE patients (*n* = 32). (**a**) SLEDAI-2k scores, and (**b**) Anti-ds-DNA antibody. Spearman’s correlation coefficient (r) and p-value (p). *p < 0.05, **p < 0.01, ***p < 0.001, ****p < 0.0001. ns: not significant

Since an alteration in the proportion of memory phenotypes was observed in the cTFH cell subsets of our patients, we further analyzed whether CM or EM cells were correlated with the level of disease activity and/or anti-dsDNA antibodies. A positive correlation between cTFH1-CM cells and disease activity was observed (*r* = 0.49, *p* < 0.01), whereas this was not the case for cTFH17-CM cells (*r* = 0.33, *p* > 0.05) (Fig. 4a). The percentage of CM in both cTFH1 and cTFH17 cell subsets was not positively related to anti-dsDNA antibody levels (cTFH1-CM; *r* = 0.28, *p* > 0.05, cTFH17-CM; *r* = 0.11, *p* > 0.05) (Fig. 4a). In addition, the EM cells in cTFH1 and cTFH17 subsets were not significantly related to scores of disease activity nor anti-dsDNA antibody levels (Fig. 4).

### SLE patients who had increased cTFH17 cells presented with aNAV and DN2 B cell expansion

**Fig. 5:**
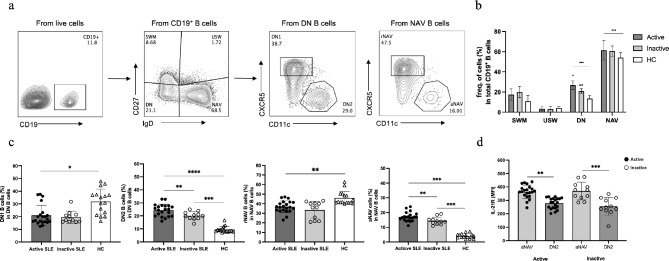
The phenotype and frequency of heterogeneous B cell subpopulations in SLE patients. (**a**) Representive gating strategy of the B cell subsets. The four independent subpopulations were gated: CD27^+^IgD^−^ switched memory (SWM), CD27^+^IgD^+^ unswitched memory (USW), CD27^−^IgD^−^ double negative (DN), and CD27^−^IgD^+^ naive B cell (USW). Then, further analysis of NAV and DN: CD11c^+^CXCR5^−^ resting naïve B cell (rNAV), CD11c^−^CXCR5^+^ activated naïve B cell (aNAV), CD11c^+^CXCR5^−^ double negative 1 (DN1) and CD11c^−^CXCR5^+^ double negative 2 (DN2). (**b**) The frequency of each B cell subset in active (*n* = 21), inactive (*n* = 11) SLE patients and in HCs (*n* = 16 ). (**c**) Frequency of DN1, DN2, rNAV, and aNAV in SLE patients and HCs. (**d**) Mean fluorescent intensity (MFI) of T cell co-stimulatory molecules (IL-21R) expressed on aNAV and DN2 B cell subset in active (*n* = 21) and inactive SLE patients (*n* = 11). Each bar represents the median and the error bar represents the interquartile range (IQR). Statistical testing was performed by one-way ANOVA test for comparing non-parametric groups; * *p* < 0.05, ** *p* < 0.01, *** *p* < 0.001, **** *p* < 0.0001, and ns: not significant

The expansion of aNAV and DN2 B cells was observed in SLE patients and the higher frequency of these B cell phenotypes was significantly related to disease activity [4, 26]. Since our data showed a positive correlation between both increased cTFH1 and cTFH17 cells, and anti-dsDNA antibodies in subjects with active SLE, we hypothesized that activation of these cTFH cells might contribute to B cell function. Thus, we sought to identify a subset of B cells that showed a positive correlation with cTFH1 or cTFH17 cell responses. The frequency of each B cell subset in SLE patients was compared to that in HCs (Fig. 5a). The frequency of each subset of B cell in SLE patients and HCs was summarized in Table 2. In active patients, both NAV B cells and DN B cells were significantly increased compared to those in HCs (Fig. 5b). However, SWM and USW B cells were not different from those in HCs (Fig. 5b).

**Table 2:** The frequency of each B cell subset in active (*n* = 21), inactive SLE (*n* = 11), and HCs (*n* = 16)

Parameters	Active SLE Median [IQR]	Inactive SLE Median [IQR]	Healthy controls Median [IQR]	*p*-value
NAV B cell	61.00 [56.55–69.85]	60.40 [56.70–66]	53.10 [50.3–56.1]	< 0.0001
DN B cell	26.90 [23.30-30.38]	21.00 [18.91–22.72]	12.90 [11.20-16.77]	< 0.0001
SWM B cell	16.20 [13.45–32.48]	20.30 [16.04–28.70]	11.20 [5.66–20.30]	ns
USW B cell	3.21 [2.16–5.01]	2.11 [1.71–4.03]	4.71 [3.02–5.15]	ns
DN2 B cell	25.11 [19.85–27.71]	20.00 [18.16–22.30]	8.55 [8.11–9.44]	< 0.001
rNAV B cell	36.37 [32.85–41.55]	36.09 [21.72–40.21]	42.55 [41.40–50.5]	< 0.0001
aNAV B cell	16.88 [14.22–19.07]	14.02 [11.92–15.78]	3.58 [2.99–5.12]	< 0.001

We further assessed the frequency of NAV and DN B cell subpopulations. In active patients, the frequency of DN1 B cells was reduced compared to HC (Fig. 5c). In contrast, the percentage of DN2 B cells were increased (Fig. 5c). In NAV B cells, a significant decrease of rNAV was found in active patients, compared to HCs (Fig. 5c). The percentage of aNAV B cells in active patients was significantly higher than in inactive and HCs (*p* < 0.001) (Fig. 5c). In addition, we observed the expression of IL-21R in aNAV and DN2 B cells. Upregulation of IL-21R molecules in B cells was defined as the MFI (mean fluorescence intensity) of SLE patients being higher than the median MFI of HCs. Since aNAV and DN2 B cells showed increased frequency in active SLE patients, we observed IL-21R expression in these B cell subsets. Significantly higher expression of IL-21R was detected in aNAV B cells in both active and inactive SLE subjects compare to DN2 B cells (Fig. 5d).

### Activation of cTFH17 cells was strongly related to the frequency of aNAV and DN2 B cells, and disease activity

**Fig. 6:**
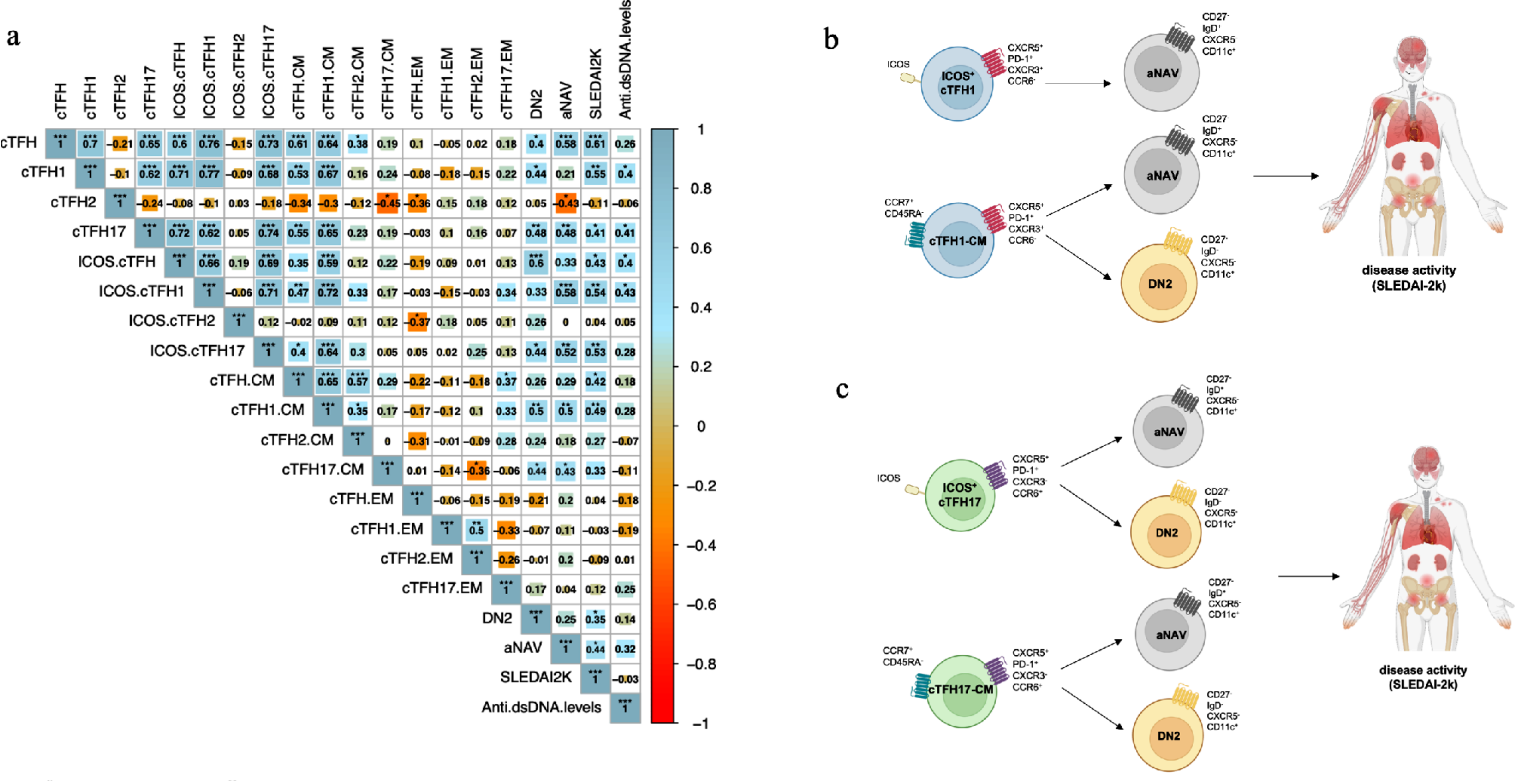
Correlation of cTFH, memory cTFH, B cells, and clinical parameters in SLE patients. **(a**) The ward.D2 hierarchical agglomerative clustering method was used to cluster correlation matrices. Spearman’s p and significance adjusted for multiple testing using the Holm method are indicated. * *p* < 0.05, ** *p* < 0.01, *** *p* < 0.001, and **** *p* < 0.0001. The coloring of the cell represents a correlation sign: blue gradient for positive correlation and red gradient for negative correlation. The size of the background squares represents the degree of correlation. Graphical summary of an association of (**b**) ICOS^+^cTFH1, cTFH1-CM, B cell subsets, and disease activity; (**c**) ICOS^+^cTFH17, cTFH17-CM, B cell subsets, and disease activity. (created with Bioreder.com/Mahidol university)

The interactions between cTFH and B cells help B cells to produce antibodies [27, 28]. We, thus, first determined the correlations of aNAV and DN2 B cells, and anti-dsDNA autoantibodies with disease activity. Expansion of aNAV (*r* = 0.44, *p* < 0.05) and DN2 (*r* = 0.35, *p* < 0.05) B cells were statistically correlated with SLEDAI-2 K scores, whereas they were not related to anti-dsDNA antibody levels (aNAV; *r* = 0.32, *p* > 0.05 and DN2 *r* = 0.14, *p* > 0.05) (Fig. 6). Further assessment of correlation between activation of cTFH cells (cTFH1 and cTFH17), and expansion of B cells (aNAV and DN2) showed that ICOS^+^ cTFH1 (*r* = 0.58, *p* < 0.001), ICOS^+^ cTFH17 cells (*r* = 0.52, *p* < 0.01) and aNAV B cell frequency were positively correlated (Fig. 6). A positive correlation between activation of ICOS^+^ cTFH17 cells and percentage of DN2 B cells was found (*r* = 0.44, *p* < 0.05) (Fig. 6). We also assessed the relationship of memory cTFH cells to B cells. A significant correlation was found of both cTFH1-CM (*r* = 0.50, *p* < 0.01), cTFH17-CM (*r* = 0.43, *p* < 0.05) cells with aNAV B cells (Fig. 6). Also, the CM cells in cTFH1 (*r* = 0.50, *p* < 0.01) and cTFH17 (*r* = 0.44, *p* < 0.05) cells were both positively correlated with DN2 B cells (Fig. 6).

## Discussion

TFH cells play an important role in the production of antibodies. Previous studies demonstrate an expansion of TFH cells in the blood of SLE patients and that these expanded cells were positively correlated with disease activity, circulating plasmablasts, and anti-dsDNA antibodies [17, 19, 29]. However, the specific subset of cTFH cells and the memory phenotype that play roles in autoantibody production and are clinically significant in SLE patients remains unclear. Here, we comprehensively analyzed frequencies and phenotypic function of cTFH cells and evaluated associations of the responses of cTFH subsets with B cells, anti-dsDNA antibodies and SLEDAI-2k scores. Our results demonstrated that cTFH cells polarized toward cTFH1 and cTFH17 cells in SLE patients. In subjects with active disease, both cTFH subsets had significantly upregulated ICOS molecules which were positively related to SLEDAI-2k score, while only ICOS^+^ cTFH1 cells were significantly correlated with anti-dsDNA autoantibody level. Characterization of memory cells in cTFH1 and cTFH17 subsets showed CM to be predominant in cTFH, while cTFH17 was in EM cells. We further determined correlations among activated cTFH, memory cTFH and B cells. The increased percentage of ICOS^+^ cTFH1 and cTFH17 had a positive correlation with an expansion of aNAV. However, only ICOS^+^ cTFH17 cells were significantly related to DN2 B cells. The presence of high percentages of CM cells in both cTFH1 and cTFH17 was positively associated with aNAV and DN2 B cell expansion. Our study demonstrated that SLE induced cTFH cell polarization towards cTFH1 and cTFH17 subsets, and these contributed to humoral immunity and pathology.

Accumulating evidence indicate that an increase in cTFH cells is involved in the pathogenesis of SLE [16, 27]. However, the roles of specific cTFH subsets in the activation of B cell responses and development of autoantibodies are poorly defined. Here, cTFH1 and cTFH7 cells were found to be significantly expanded and activated in patients with active SLE, as reflected in their disease activity scores. These results are similar to previous reports of significant increases in the proportion of circulating activated TFH-Th1-like cells [18], and frequency of cTFH17 cells in both French and Chinese patients compared to healthy control subjects [19, 20]. We did not find a positive correlation between the percentage of cTFH2 cells and disease activity. In contrast, cTFH2 cells and SLEDAI-2k score were significantly correlated in previous study [15, 19]. Regarding the relationship between activated cTFH1 cells and the SLEDAI-2k scores of patients with active disease, it was clearly demonstrated that IFN-γ influenced the function of macrophages and dendritic cells to produce inflammatory cytokines [30], and these activated cells secrete B lymphocyte-stimulating factor (BLyS) which support B cell survival [31]. Thus, our data might explain the effect of IFN-γ secreted by cTFH1 cells to induce the function of innate immune cells resulting in the development of inflammation and activating GC B cells for IgG autoantibody secretion, together contributing to SLE pathogenesis. Regarding the relationship of cTFH17 cells to SLEDAI-2k scores, it may be that activated ICOS^+^ cTFH17 cells produce IL-17 cytokine that induces the recruitment of monocytes and neutrophils to peripheral tissues [32]. They may also participate in the activation of B cells producing autoantibodies [33]. leading subsequently to the local inflammation and tissue damage of SLE. Besides effector T helper (TH) 17 cells, cTFH17 cells may cooperate with TH17 cells and increase disease activity in SLE patients, consistent with previous evidence of an association between IL-17 cytokine and SLEDAI-2k scores [29]. Our findings highlighted the function of cTFH1 and cTFH17 cells in SLE pathogenesis. Elucidation of the functions of these cells in SLE pathogenesis would be very useful in improving the strategies of targeted therapeutics.

Memory T cells are implicated in the pathogenesis of SLE [34]. Here, memory cTFH cells were characterized as CM or EM cells by distinct homing capacity and effector function [25]. In our subjects with active disease, a significant increase in CM phenotype was found in both cTFH1 and cTFH17 cells, compared to those in inactive subjects. However, only cTFH17-EM cells were increased in active SLE. The CM/EM cell ratio showed an alteration of cTFH17-CM cells into the effector phase, while memory cTFH1 cells predominantly persisted in the CM phenotype. In addition, the frequency of cTFH1-CM cells was positively related to SLEDAI-2 K score, whereas this did not occur with the cTFH17-CM cell population. Memory cTFH1 and cTFH17 cells (both CM and EM) were not correlated with anti-dsDNA antibody levels. A possible explanation for these results is that SLE induces cTFH1 and cTFH17 cell polarization, which could develop memory cells in both TFH subsets and result in increased CM cells in cTFH1 and cTFH17 cells in peripheral blood of active patients. The inflammation and cytokine milieu (IL-17 and IL-22) of active SLE patients could strongly induce differentiation of cTFH-17-CM toward effector phase and lead to migration into inflamed organs. In contrast to memory cTFH17 cells, the frequency of cTFH1-CM cells was high and positively related to SLEDAI-2k score. Our data indicated that different stimuli were involved in the generation of the effector phase of cTFH1 and cTFH17 cells. Future studies in the mechanisms of memory cTFH17 responses, differentiation of EM cells in this cTFH cell subset, as well as its contribution in helping B cells to secret autoantibodies, are needed.

It is reported that cTFH cells play a role in the generation of GC B cells and antibody production [27, 28]. To gain more knowledge of the cooperation between these two cells in producing autoantibodies in SLE, we first determined the frequencies of each B cell subset in subjects whose cTFH1 and cTFH17 cells were expanded. The frequencies of aNAV and DN2 B cells were found to be elevated and correlated with disease activity scores. This was consistent with previous studies that showed expansion of aNAV and DN2 B cells, and the critical significance of these B cell subsets in SLE pathogenesis [4, 35, 36]. Next, we assessed the relationship between cTFH and B cells. The ICOS^+^ cTFH1 and ICOS^+^ cTFH17 cells were both positively related to aNAV B cells, while only ICOS^+^ cTFH17 cells had a significant correlation with DN2 B cells. Moreover, positive correlations between CM cells (cTFH1 and cTFH17) and both aNAV and DN2 cells also were observed. In our SLE subjects, cTFH and B cell interactions were demonstrated. The frequency of TFH2 cells was correlated with double negative (CD27^−^IgD^−^CD19^+^) B cells or plasmablasts. However, in a study of cTFH17 cells, a positive correlation between the frequency of cTFH17 cells and plasmablasts was not detected [19]. These previous data indicate that TFH2 cells help B cells to produce autoantibodies. However, it remains unclear whether cTFH1 or cTFH17 cells are involved in the T-dependent activation of B cells. In our subjects, cTFH17 cell activation was significantly correlated with the frequencies of aNAV and DN2 B cells, which in turn upregulated IL-21R (aNAV = 22% and DN2 = 12.5%). Thus, the cooperation between cTFH17 cell and aNAV or DN2 B cell might drive B cells to produce autoantibodies via IL-21 signaling. This is consistent with the previous report that CD4^+^ T cells and IL-21 cytokine enhanced aNAV B cell differentiation into auto-ASCs and produced anti-DNA antibodies [37]. Further studies of cTFH17 function in helping aNAV and DN2 B cells to generate humoral immunity may potentially benefit the clinical management of SLE.

There are shortcomings in this study. First, a larger sample size with multiple measures of clinical status (e.g., disease activity, organ involvement, and treatment profile) is required to further demonstrate the functional role of cTFH cells and their association with SLE pathogenesis. Second, cTFH cell subsets were phenotyped based on levels of CXCR3 and CCR6 expression, as done in previous studies [12, 15, 38]. We did not exclude FOXP3^+^ follicular regulatory T (TFR) cells before identifying the cTFH cell subset. Thus, our study could not explain TFH/TFR dysregulation in humoral immunity. Third, secretory cytokine profiles of each subset of cTFH cells (IFN-γ, IL-4, IL-10, IL-17, and IL-21) were not determined (no deep phenotyping). Such data would add more understanding to the function of each cTFH cell subset. Last, the kinetic responses of cTFH1 and cTFH17 cells were not determined. A cohort study following these responses could be useful for the application of these cTFH subsets as biomarkers for monitoring disease activity in SLE.

## Conclusion

This study demonstrated that cTFH cells in SLE patients were skewed toward cTFH1 and cTFH17 cell responses and related to aNAV B or DN2 B cell responses. Together these led to involvement in the development of anti-dsDNA autoantibodies and SLE pathogenesis. Deep knowledge of the cooperative function of cTFH1 or cTFH17 with these two B cell subsets provides novel strategies for inhibition of autoantibody production in SLE patients.

## Electronic supplementary material

Below is the link to the electronic supplementary material.

Supplementary Material 1

Supplementary Material 2

Supplementary Material 3

Supplementary Material 4

Supplementary Material 5

## Data Availability

The datasets used and/or analyzed during the current study are available from the corresponding author upon reasonable request.

## Abbreviations

aNAV: Activated naïve
Anti-dsDNA: Anti-double stranded DNA
ASCs: Antibody-secreting cells
ACR: American College of Rheumatology
C3: Complement 3
C4: Complement 4
DN1: Double negative 1
DN2: Double negative 2
ESR: Erythrocyte sedimentation rate
FITC: Fluorescein isothiocyanate
HCs: Healthy controls
IFN-γ: Interferon-γ
IL: Interleukin
PBMCs: Peripheral blood mononuclear cells
PerCP/Cy5.5: Peridin-chlorophyll protein/ Cyanine 5.5
PE: Phycoerythrin
R: Correlation coefficient
R848: Resiquimod
SLE: Systemic lupus erythematosus
SLEDAI-2k: SLE disease activity index-2 K
SLICC: Systemic lupus erythematosus international collaborating clinics
SWM: Switched memory B cells
USW: Unswitched memory B cells
