# Supplementary material for: Activation of circulating TFH17 cells associated with activated naive and double negative 2 B cell expansion, and disease activity in systemic lupus erythematosus patients
Source: Arthritis Res Ther. 2024 Sep 11;26:159. doi: 10.1186/s13075-024-03394-7 (PMC11389436; doi:10.1186/s13075-024-03394-7)
Supplement: Supplementary file 1 — Supplementary Material 1 [file 13075_2024_3394_MOESM1_ESM.pdf]

Supplementary Table S1 Demographic, laboratory, and clinical characteristics of SLE patients and healthy control recruited in this study

Category	Feature	SLE patients (n = 32)	Healthy control (n=16)
Demographic	Age, year (mean ± SD)	32.45 ± 12.19	27.5 ± 5.76
	Gender, male, n (%)	4/32 (12.5 %)	6/16 (37.5%)
	Female, n, (%)	28/32 (87.5 %)	10/16 (62.5%)
	Disease duration, week (#)	35 (2-96)	N/A
	Lupus nephritis, LN (#)	22/32 (68.75%)	N/A
	Non-LN (#)	10/32 (31.25%)	N/A
Laboratory parameters	SLEDAI-2K (#)	3 (0-10)	N/A
	Anti-dsDNA positive No (%)	9/32 (28.13 %)	N/A
	ESR, mm/h (#)	33 (1-94)	N/A
	Serum albumin (#)	37.84 (27.5-43.8)	N/A
	C3, g/L (#)	1.114 (0.40-5.32)	N/A
	C4, g/L (#)	0.248 (0.06-0.57)	N/A
Clinical manifestations	Vasculitis, n (%)	1 (3.12%)	N/A
	Arthritis, n (%)	0 (0 %)	N/A
	ESRD, n (%)	1 (3.12 %)	N/A
	APS, n (%)	1 (3.12 %)	N/A
	Multi-target, n (%)	8 (25 %)	N/A
Treatment	Prednisolone, n (%)	29/32 (90.63%)	N/A
	Antimalarial, n (%)	25/32 (78.12%)	N/A
	Azathioprine, n (%)	6/32 (18.75%)	N/A
	Mycophenolate, n (%)	16/32 (50%)	N/A
	Methotrexate, n (%)	8/32 (25%)	N/A

SLEDAI-2K: Systemic Lupus Erythematosus Disease Activity Index 2000; Anti-dsDNA: Anti-double stranded DNA antibody; WBC: White Blood Cell counts; ESR: Erythrocyte Sedimentation Rate; C3: Complement 3; C4: Complement 4; ESRD: End Stage Renal Disease; APS: Antiphospholipid syndrome. # = [median (range)]; N/A: not available.
