# Supplementary material for: Activation of circulating TFH17 cells associated with activated naive and double negative 2 B cell expansion, and disease activity in systemic lupus erythematosus patients
Source: Arthritis Res Ther. 2024 Sep 11;26:159. doi: 10.1186/s13075-024-03394-7 (PMC11389436; doi:10.1186/s13075-024-03394-7)
Supplement: Supplementary file 3 — Supplementary Material 3 [file 13075_2024_3394_MOESM3_ESM.pdf]

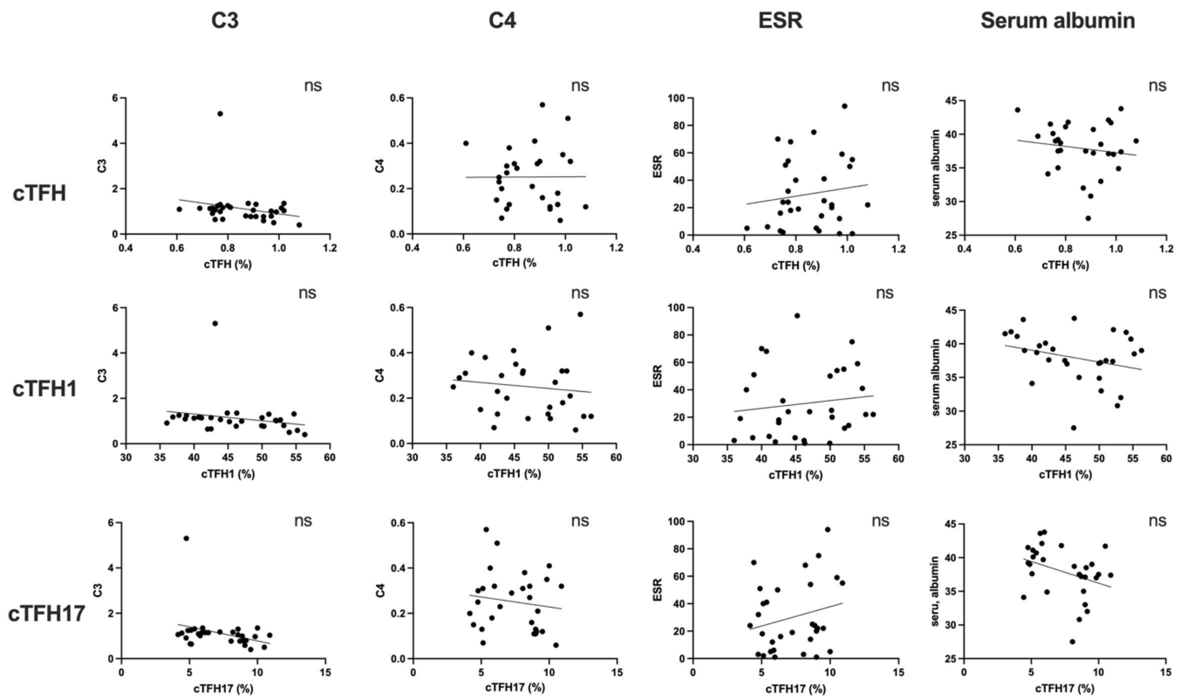

Supplementary Fig 3. The correlation of cTFH subset, complement C3, C4 levels, ESR, and serum albumin in total SLE patients (n = 32). Spearman's rank correlation coefficients are shown; ns: not significant.
