# Supplementary material for: Activation of circulating TFH17 cells associated with activated naive and double negative 2 B cell expansion, and disease activity in systemic lupus erythematosus patients
Source: Arthritis Res Ther. 2024 Sep 11;26:159. doi: 10.1186/s13075-024-03394-7 (PMC11389436; doi:10.1186/s13075-024-03394-7)
Supplement: Supplementary file 4 — Supplementary Material 4 [file 13075_2024_3394_MOESM4_ESM.pdf]

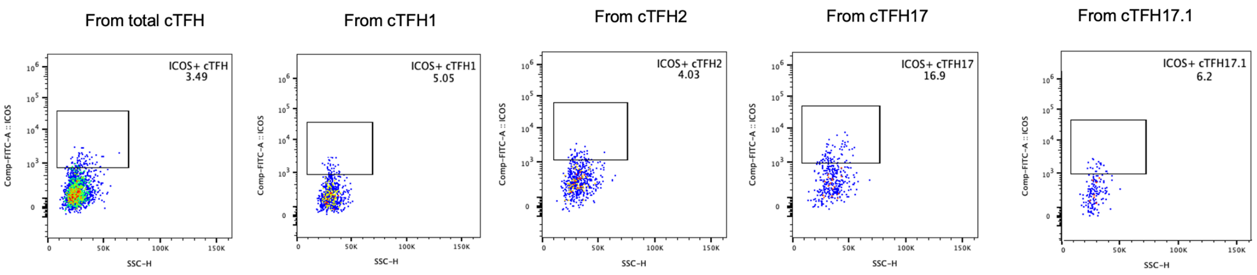

Supplementary Fig 2. The gating strategy of the ICOS expression in total cTFH, cTFH1, cTFH2, cTFH17, and cTFH17 cells from SLE patients.
