# Supplementary material for: Activation of circulating TFH17 cells associated with activated naive and double negative 2 B cell expansion, and disease activity in systemic lupus erythematosus patients
Source: Arthritis Res Ther. 2024 Sep 11;26:159. doi: 10.1186/s13075-024-03394-7 (PMC11389436; doi:10.1186/s13075-024-03394-7)
Supplement: Supplementary file 2 — Supplementary Material 2 [file 13075_2024_3394_MOESM2_ESM.pdf]

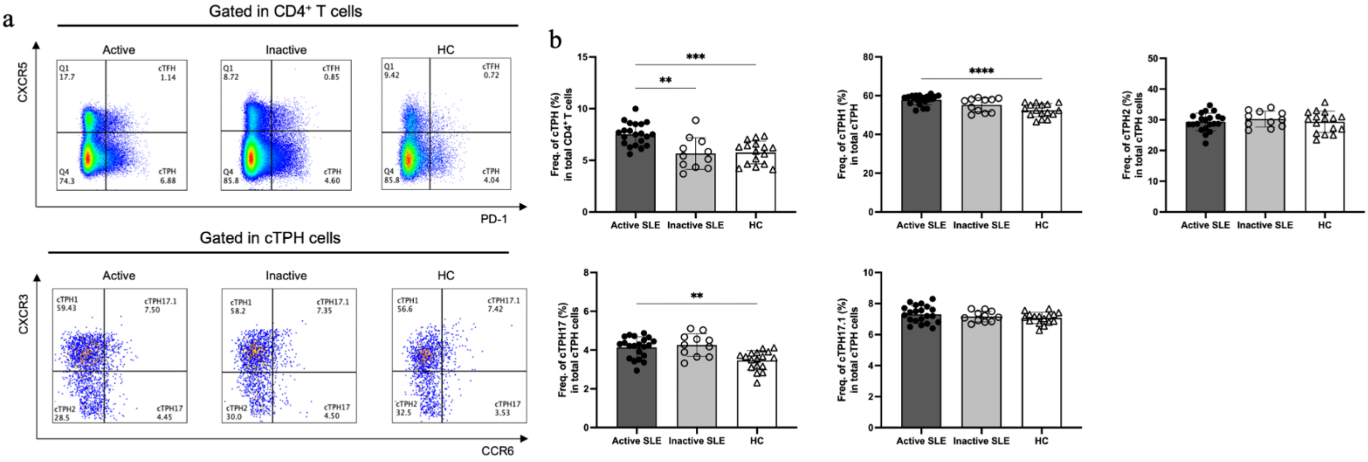

Supplementary Fig 1. (**a**) Representative flow cytometry gating strategy of cTPH cells ($CXCR5^-$ $PD\text{-}1^+$) from SLE and HC subjects. Three subsets were gated: cTPH1 ($CXCR3^+CCR6^-$), cTPH2 ($CXCR3^-CCR6^-$), and cTPH17 ($CXCR3^-CCR6^+$) (**b**) The frequency of total cTPH cells and their subsets in active (n=21) and inactive SLE patients (n=11) compared to HCs (n = 16). Bars represent the median with interquartile range; *p* values were determined by the one-way ANOVA: ** $p < 0.01$, *** $p < 0.001$, and **** $p < 0.0001$. ns: not significant
